# Particle Beam Therapy for Intrahepatic and Extrahepatic Biliary Duct Carcinoma: A Multi-Institutional Retrospective Data Analysis

**DOI:** 10.3390/cancers14235864

**Published:** 2022-11-28

**Authors:** Hideya Yamazaki, Takuya Kimoto, Motohisa Suzuki, Masao Murakami, Osamu Suzuki, Masaru Takagi, Norio Katoh, Takeshi Arimura, Takashi Ogino, Hiroyuki Ogino

**Affiliations:** 1 Department of Radiology, Graduate School of Medical Science, Kyoto Prefectural University of Medicine, Kyoto Kawaramachi Hirokoji, Kamigyo-ku, Kyoto 602-8566, Japan; 2 Department of Radiation Oncology, Southern TOHOKU Proton Therapy Center, Koriyama 963-8052, Japan; 3 Osaka Heavy Ion Administration Company, Otemae, Chuo-ku, Osaka 540-0008, Japan; 4 Proton Therapy Center, Sapporo Teishinkai Hospital, Sapporo 065-0033, Japan; 5 Department of Radiation Oncology, Hokkaido University Faculty of Medicine, Sapporo 060-8648, Japan; 6 Medipolis Proton Therapy and Research Center, Ibusuki, Kagoshima 891-0304, Japan; 7 Department of Radiation Oncology, Nagoya Proton Therapy Center, Nagoya City University West Medical Center, Nagoya 462-8508, Japan

**Keywords:** biliary duct carcinoma, extrahepatic bile duct carcinoma, intrahepatic duct carcinoma, particle beam therapy

## Abstract

**Simple Summary:**

We examined the outcome of patients with biliary duct carcinoma treated with particle beam therapy, which has a potential advantage to be prescribed at a higher dose. The median survival time (MST) was 21 months in the total population, and were 20 and 23 months for extrahepatic BDC and intrahepatic BDC, respectively. A higher radiation dose EQD2 ≥ 67 Gy improved OS in extrahepatic BDC. PT showed good efficacy for BDC, both eBDC and iBDC, with a low incidence of severe toxicity.

**Abstract:**

To examine the efficacy and toxicity of particle beam therapy (PT) for biliary duct carcinoma (BDC) and compare the outcomes between extrahepatic BDC (eBDC) and intrahepatic BDC (iBDC). We analyzed multi-institutional data from May 2009 to December 2019. The primary endpoint was overall survival (OS), and the secondary endpoints were local control (LC), progression-free survival (PFS) and toxicity. We included 150 patients with unresectable BDC treated with PT using a median prescribed dose of 70.2 GyRBE (range, 44–77 GyRBE) in 25 fractions (range, 10–38 fractions). With a median follow-up of 13.0 months, median survival time (MST) was 21 months, and 2-year OS was 44.8%. For eBDC and iBDC, the MSTs were 20 and 23 months, respectively. Two-year PFS and LC rates were 20.6% and 66.5%, respectively. Vascular invasion, prescribed dose and serum tumor marker level (carcinoembryonic antigen: CEA) were identified as poor prognostic factors for OS. A higher radiation dose EQD2 ≥ 67 Gy showed superior OS, with a hazard ratio of 0.341. The radiation dose of PT is an important predisposing factor for overall survival. The MST for patients with eBDC given a higher radiation dose was 25 months, compared to 15 months for those given the lower dose and 23 months for patients with iBDC (all iBDC given higher doses). iBDC and eBDC duct carcinomas showed equivalent outcomes with PT, especially when treated with a high radiation dose. In detailed analysis, baseline CEA level in iBDC, and radiation dose and GTV in eBDC were statistically significant predicators for OS. Acute and late toxicity grade ≥3 occurred in 2.2% and 2.7% of patients, respectively, including two late grade-5 toxicities. In conclusion, PT showed good efficacy for BDC, both eBDC and iBDC, with a low incidence of severe toxicity.

## 1. Introduction

Biliary duct carcinoma (BDC) comprises a heterogeneous population, including intrahepatic BDC (iBDC) and extrahepatic BDC (eBDC; perihilar, distal cholangiocarcinoma, and gallbladder cancer). These are rare malignancies in most high-income countries, but represent a major health problem in endemic areas [1,2]. 

Surgery is considered the only curative procedure; however, few patients can undergo upfront resection because of local disease progression [1,3]. For unresectable cases, the standard treatment is systemic chemotherapy, i.e., gemcitabine and cisplatin; however, the prognosis is poor, with a median survival of approximately one year [1,4]. Several studies have suggested that radiotherapy (RT) could improve tumor control and survival, but this lacked a high level of evidence [1,4,5]. One reason for this was the limited prescribed dose of conventional RT, which is restricted by adjacent organs at risk (i.e., intestine, stomach and liver)—this results in tumor progression inside the irradiation field. Technical advancements in RT, stereotactic radiotherapy (SBRT), intensity-modulated radiotherapy (IMRT), respiratory gating and image guidance with computer tomography have enabled the delivery of larger doses to the tumor without elevating the dose in surrounding normal tissues [5,6,7,8]. Additionally, particle beam therapy (PT) using protons or carbon ions has emerged as a highly promising procedure. PT has an advantageous physical property over radiotherapy with photons, as a spread-out Bragg peak (SOBP) offers superior dose distribution for the target volume [9,10,11,12]. Several studies have reported outcomes of PT for BDC [9,10,11,12]. 

Evidence indicates distinctly different characteristics between iBDC and eBDC, including differing molecular profiles [1,2,13]. Different definitions and statistics were performed between iBDC (one of the liver cancers [14]) and eBDC (an independent category) as separate entities, although some data are available for comparing the differences between iBDC and eBDC for PT [9,10,11,12]. Therefore, we conducted a comparative study of eBDC and iBDC. 

This study aimed to examine the efficacy and toxicity of PT for BDC, and compare the outcomes between eBDC and iBDC.

## 2. Materials and Methods

This retrospective study included patients with non-metastatic BDC treated with PT at 6 institutions between May 2009 and June 2019. The inclusion criterion was unresectable extrahepatic cholangiocarcinoma unsuitable for curative surgical treatment (patients who refused surgery were deemed unresectable). From 185 patients during initial registration, 35 were excluded for the following reasons: previous surgery or planned surgery (*n* = 11) and recurrence (*n* = 24). We included 150 patients in the analysis (Table 1). 49 out of 53 patients with jaundice received stenting after endoscopic or percutaneous drainage.

The most frequently used schedules were 72.6 GyRBE/22 fractions (*n* = 25), 76 GyRBE/20 fraction and 76 GyRBE/38 fraction (*n* = 17) (Table S1). One patient stopped treatment for biliary tract infection at 44 Gy/22 fraction. The major systemic therapy agent for concurrent therapy was gemcitabine or TS-1, and was a combination of both cisplatin and gemcitabine for the neoadjuvant (adjuvant) setting. Details of the treatment in each institution have been described elsewhere [9,10,11,12]. In brief, 144 patients were treated with a passive scatter broad beam, and 4 patients with spot scanning. A respiratory gating system (Anzai Medical, Tokyo, Japan) was used in several institutions. Daily image guidance/motion management was performed using gold marker and pretreatment imaging (MVCT, Orthogonal kVX ray, etc.) in several institutions. 

All patients were staged according to the 7th edition of the Tumor—Node—Metastasis Staging System (International Union Against Cancer, 2009). We analyzed overall survival (OS) as the primary endpoint. Progression-free survival (PFS), local control rate (LC) and toxicity were analyzed as secondary endpoints. This multicenter retrospective data accumulation study was approved by the institutional review board (Kyoto Prefectural University of Medicine; ERB-C-1747-2) and each participating institution. The study protocol was performed according to the principles of the Declaration of Helsinki. 

Equivalent 2-Gy fractions (EQD2 = *n* × *d*((α/β) + d)/((α/β) +2): *n* = number of treatment fractions: *d* = dose per fraction in Gy, α/β = 10) were used for the radiation dose estimation. 

Adverse events were classified according to the National Cancer Institute Common Terminology Criteria for Adverse Events, version 4.0. Acute toxicities were defined as occurring during PT or within 90 days after PT completion, and late toxicities occurred after 90 days. 

### Statistical Analyses

StatView 5.0 and EZR stat package15 was used for statistical analyses [15]. Percentages were analyzed using chi-square tests, and Student’s *t*-tests were used for normally distributed data. Mann–Whitney U-tests for skewed data were used for comparisons. The Kaplan–Meier method was used to analyze OS, PFS and LC. The time of the event was determined from the start of PT. Cut-off values were set at the median or mean value if they were not specified. For GTV, CTV, PTV and baseline CEA level, we used ROC analysis to define the cut-off values. We used 67 Gy in EQD2 (≈ 80.5 Gy in BED10; α/β = 10) as a cut-off value for the prescribed dose according to the previous study [16]. Cox’s proportional hazard model was used for uni- and multivariate analyses (variable *p* ≤ 0.2 was entered into multivariate analysis). *p* < 0.05 was considered statistically significant.

## 3. Results

### 3.1. Patient Characteristics

A total of 150 patients underwent PT for nonmetastatic fresh BDC between 2009 and 2019. Detailed patient, tumor and treatment characteristics are shown in Table 1. The median age of all patients was 74 years (range: 50–94 years). Here, 64.6% of patients were male, and 94.0% had a good performance status, with 0–1. The median tumor diameter was 4.0 cm (range: 1.0–15.3 cm) and the median prescribed dose was 70.2 Gy (range: 44–77 Gy) in 25 fractions (range: 10–38 fractions). iBDC had greater tumor volume, less frequent lymph node involvement and wider distance between the tumor and gastrointestinal (GI) tract than in eBDC. Patients with iBDC underwent a higher dose of radiotherapy and less frequent concurrent chemotherapy than those with eBDC. No background difference was found in patients who underwent proton and carbon (Table S2).

### 3.2. Local Control, Progression-Free Survival, Failure Pattern and Overall Survival Rate in Total Population

**Figure 1:**
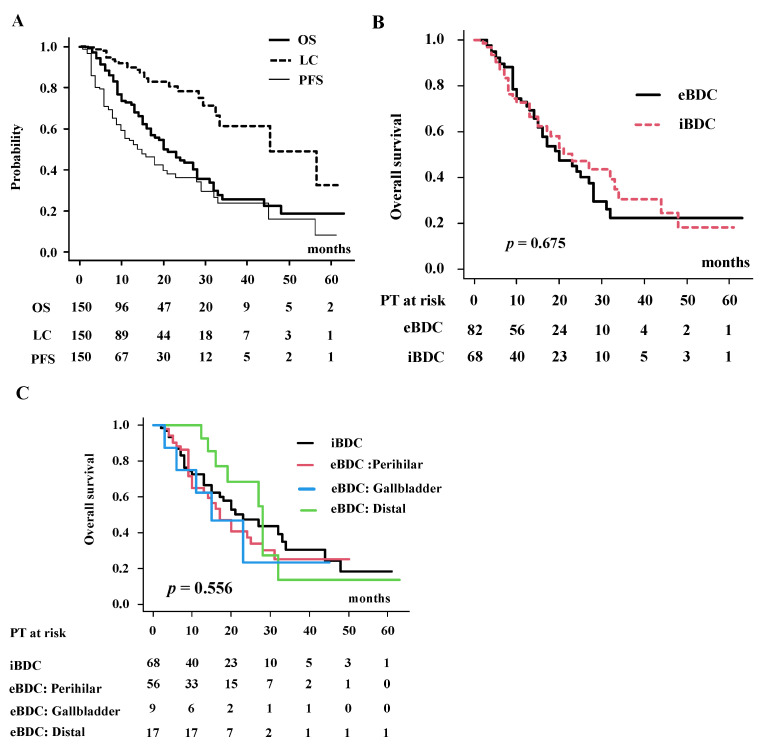
Overall survival rate (OS), progression-free survival rate (PFS) and local control (LC). (**A**) Overall survival rate (OS), progression free survival rate (PFS) and local control rate (LC). (**B**) OS between extrahepatic biliary duct carcinoma (eBDC) and intrahepatic biliary duct carcinoma (iBDC). (**C**) OS according to primary location.

With a median follow-up of 13.0 months, median survival time (MST) was 21 months (95% confidence interval (CI): 17–28 months), and 1- and 2-year OS were 72.8% (95% CI: 64.2–79.6%) and 44.8% (95% CI: 34.8–54.3%) (Figure 1A). For iBDC and eBDC, MST was 23 months (95% CI: 15–34 months) and 20 months (95% CI: 15–28 months) (*p* = 0.675, Figure 1B), respectively. One- and 2-year OS were 72.9% (95% CI:61.2–81.6%) and 42.6% (95% CI: 29.2–55.4%) for eBDC and 72.6% (95% CI: 59.2–82.3%) and 47.3% (95% CI: 32.4–60.8%) for iBDC, respectively. For detailed location, MST was 17 months (95% CI: 10–25 months), 15 months (95% CI: 3 months-NA) and 28 months (95% CI: 16–32 months) for perihilar, gallbladder and others, respectively (Figure 1C). Two-year survival rates were 37.4% (95% CI:22.0–52.8%) for perihilar, 68.6% (95% CI:35.9–87.0%) for distal and 23.4% (95% CI: 0.1–61.6%) for gallbladder.

**Figure 2:**
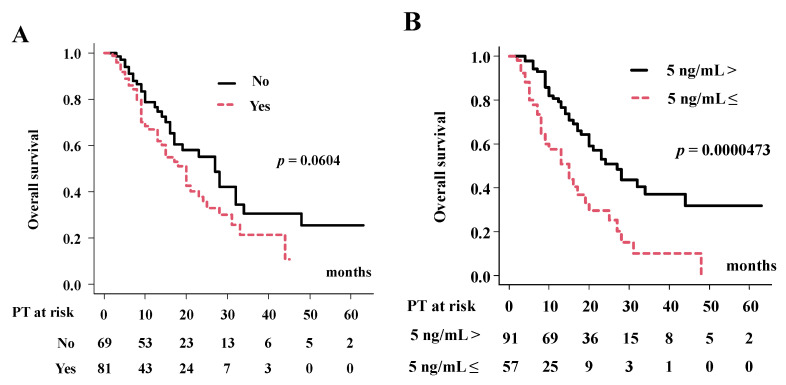

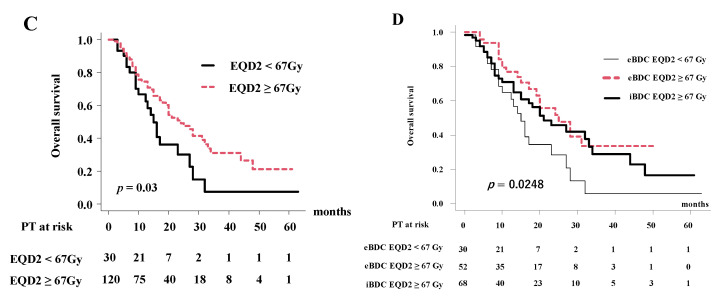
Influential factors for overall survival rate. (**A**) OS according to Vascular invasion. (**B**) OS according to pretreatment CEA level. (**C**) OS according to radiation dose. (**D**) OS according to radiation dose and primary location of tumor.

As shown in Table 2, predictors of poor OS in the univariate analysis included vascular invasion, serum level of the tumor marker carcinoembryonic antigen (CEA) and prescribed dose. In multivariate Cox regression analysis (Table 2), vascular invasion (hazard ratio (HR) = 2.26, 95% CI: 1.24–4.11, *p* = 0.007), CEA level (HR = 3.18, 95% CI: 1.83–5.52, *p* < 0.0001) and prescribed dose (HR = 0.371, 95% CI:0.19–0.72, *p* = 0.003) had significant influences on OS. Patients with vascular invasion had a 2-year OS of 35.5%, while patients without had 55.2% (Figure 2A, *p* = 0.06). Patients with a higher CEA level ≥ 37 ng/mL had a 29.5% 2-year OS, whereas patients with CEA level < 37 ng/mL were 50.9% (Figure 2B, *p* = 0.0000473). Patients treated with higher prescribed doses EQD2 ≥ 67 Gy showed a 49.1% 2-year OS, while those treated with EQD2 < 67 Gy was 30.1% (Figure 2C, *p* = 0.030). When stratifying eBDC and iBDC by prescribed dose, MST (and 2-year OS) was 15 months (30.1%) for eBDC treated with prescribed doses EQD2 < 67 Gy; 25 months (51.7%) for eBDC given EQD2 ≥ 67 Gy; and 23 months (47.3%) for iBDC given EQD2 ≥ 67 Gy (*p* = 0.0246 among 3 groups and *p* = 0.025 between EQD2 ≥ 67 Gy and EQD2 < 67 Gy in eBDC, Figure 2D).

LC was 89.7% (95% CI:82.4–94.1%) at 1 year and 78.2% (95% CI:66.7–86.2%) at 2 years (Figure 1A). Clinical target volume (CTV) (larger than 75 cm^3^) (HR = 3.327, 95% CI: 1.30–8.47, *p* = 0.011, Figure S1A) and prescribed dose (EQD2 ≥ 67 Gy) (HR = 0.341, 95% CI: 0.140–0.833, *p* = 0.018, Figure S1B) correlated with local control in multivariate analysis (Table S3).

Median PFS was 14 months (95% CI: 10–20 months) (Figure 1A), while 1- and 2-year PFS were 54.1% (95% CI:44.9–62.5%) and 35.8% (95% CI: 26.0–45.7%), respectively. CEA level was the only statistically significant prognostic factor identified (HR = 2.36, 95% CI:1.45–3.81, *p* = 0.0004) (Table S4, Figure S1C).

The major sites of progression were local (*n* = 29, 19.3%), lymph nodes (*n* = 12, 8.0%), intrahepatic failure outside irradiated area (*n* = 29, 19.3%) and distant metastases (*n* = 26, 17.3%) (Table 3).

### 3.3. Detailed Analysis of Overall Survival Rate in eBDC and iBDC

**Figure 3:**
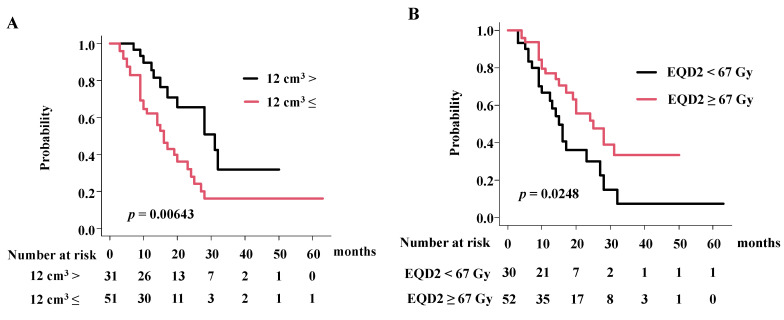
Influential factors for overall survival rate. (**A**) OS according to GTV in eBDC. (**B**) OS according to radiation dose in eBDC.

Patients with small GTV < 12cm^3^ showed a superior 2-year overall survival rate of 65.4% (41.5–81.4%), compared with patients with a large GTV with an overall survival rate of 28.0% (13.6–44.3%) at 2 years, respectively (Figure 3A, *p* = 0.00643; hazard ratio 2.30, 95% CI = 1.14–4.66–10.75, *p* = 0.01; Table 4) in eBDC. Patients with a higher prescribed dose showed a superior 2-year overall survival rate of 51.7% (33.1–67.5%), compared with patients treated with a lower prescribed dose with an overall survival rate of 30.0% (12.9–49.4%) at 2 years, respectively (Figure 3, *p* = 0.0248; hazard ratio 0.45, 95% CI = 0.24–0.87, *p* = 0.018; Table 4).

**Figure 4:**
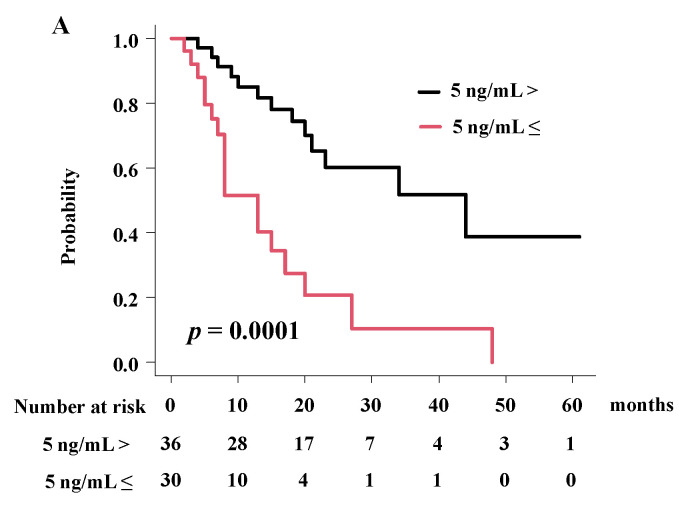
Influential factor for overall survival rate in iBDC. (**A**) OS according to baseline CEA level in iBDC.

Patients with a lower baseline CEA level showed a superior 2-year overall survival rate of 60.3% (38.8–76.3%), compared with patients treated with a higher baseline CEA level with an overall survival rate of 20.6% (5.6–42.0%) at 2 years, respectively (Figure 4, *p* = 0.0001; hazard ratio 4.08, 95% CI = 1.93–8.63, *p* = 0.0002; Table 5) in iBDC.

### 3.4. Toxicity

Acute adverse reactions of grade 3 bile duct stasis occurred in 2 patients (2/150 = 2.2%; Table 6). Cholangitis grade 1–2 occurred in 14 patients after PT. Late toxicities, grade ≥ 3, occurred at 11, 4, 9, 44 months after PT in 4 patients (2.7%). Here, two lethal toxicities were reported. A 68-year-old male with Child–Pugh A iBDC (cT4N0, tumor diameter 8.8 cm, Gross tumor volume (GTV) 322 cm^3^, CTV 514 cm^3^, distance between intestine <1 cm) received 74 GyRBE/34 fractions of proton therapy and resulted in a complete response (CR); however, he showed duodenal perforation and subsequent liver failure grade 5 44 months later. Next, a 77-year-old male with eBDC (distal bile duct, cT1N0 GTV 7.65 cm^3^, CTV33.7 cm^3^) underwent 61.6 GyRBE/28 fractions and achieved CR, but showed lethal duodenal bleeding (grade 5) 11 months later. 

## 4. Discussion

The purpose of this study was to evaluate the efficacy and toxicity of PT for BDC and compare the outcomes between eBDC and iBDC. To the best of our knowledge, this is one of the largest series of outcome reports on PT-treated BDC. Our study found that PT showed good efficacy with a low frequency of severe toxicity. Moreover, this is first study to report the importance of a higher radiation dose related to improved outcomes in separated eBDC population and equivalent outcomes between iBDC and eBDC, especially in patients treated with higher radiation doses, as demonstrated.

Radiation dose escalation had been explored for improving the outcomes of several hepatobiliary cancers [16,17,18,19]. In the 20th century, Crane et al. found that EBRT dose (30 Gy, 36–50.4 Gy and 54–85 Gy) is dose-dependent with median time to local progression (9 vs. 11 vs. 15 months), and no significant increase in toxicity [17]. However, dose escalation using conventional 3D-CRT is a difficult task, due to the accompanying increased toxicity to adjacent organs. The proximity of BDC to the bowel limits the ability to escalate the radiation dose to above 55 Gy without severe toxicity [5,19,20]. Brachytherapy has been employed to elevate the irradiated dose without elevating the irradiation of adjacent normal tissues [21,22,23]. Brachytherapy improved local tumor control near the bile duct, which increased stent patency; however, this did not translate to longer survival in the entire population [24].

In recent years, several advanced radiotherapy techniques, including SBRT and IMRT, have been introduced for treating BDC [5]. The SBRT technique enabled us to deliver a higher dose than conventional radiotherapy, resulting in improved local control, especially in lung and liver diseases [25]. However, increasing radiation dose also caused severe elevated radiotherapy-related adverse events adjacent to the target volume, i.e., the gastrointestinal tract. Lee et al. reported outcomes of SBRT (MST of 13 months) with a frequency of late toxicity around 10–20% in a systematic review [26]. The IMRT technique may therefore be an alternative to reduce normal tissue toxicity [5], with reported 45–100% LC and 58–81% 1-year survival rates [5].

Tao et al. demonstrated that dose escalation BED > 80.5 Gy10 (≃67 Gy in EQD2, proton or photon) for iBDC improved OS (73% vs. 58% 2-year OS rate) [16]. They increased the doses of radiation delivered to the tumor using a smart simultaneous integrated boost (a dose of 100 Gy in 25 fractions into the center of the tumor), and integrated protection (GTV dose does not overlap with planning risk volume) technique with hypofractionation [16]. PT has the distinct characteristic of rapid dose off; a lack of exit dose theoretically offers a higher radiation dose without elevating normal liver dose (low rates of grade 3 toxicity and/or worsening hepatic function). Hong et al. reported a 2-year survival rate of 46.5% for iBDC obtained in a prospective multicenter study of proton beam therapy and 7.7% grade ≥3 toxicity [20]. These results imply that PT could have an advantage over photons, especially in iBDC [18,19,20]. Our data of a 47.3% 2-year survival rate for iBDC concurred with their result.

There are differing characteristics between iBDC and eBDC, not only in the anatomical position of the tumor, but also in biological behavior [13]. Kang et al. reported differences in outcomes among BDC by location in the Korean population, where the highest incidence of BDC was reported. The 5-year relative survival rate was highest in the ampulla of Vater (48.5%), followed by the gallbladder (28.5%) and other sites of eBDC (19.9%) and iBDC (10.8%) [27]. Their data included all populations with or without treatment, and the difference was apparent among BDC locations. In general, iBDC showed poorer outcomes than eBDC; however, in dose-escalated radiotherapy series such as PT, iBDC did not show an inferior outcome to eBDC [9,10,11,12,20]. As eBDC is located in close proximity to the bowel, PT dosage was limited, and was difficult to elevate the tumor dose. In our cohort, all patients with iBDC could receive a higher prescribed dose of EQD2 ≥ 67 Gy, whereas 64.6% (53/82) of eBDC received a higher dose (78.5% in hilar, 17.6% in other and 55.5% in gallbladder). From the literature, PT had an MST of 23–24 months [9,10,11,12,20] in iBDC and 12.6–23 months for eBDC [12,28]. Our data concurred with the previous finding that similar MSTs of 20 months in eBDC (25 months for higher radiation dose EQD2 ≥ 67 Gy vs. 15 months with lower radiation dose EQD2 < 67 Gy) and 23 months (with higher radiation dose) in iBDC were found (Figure 2).

Elganainy et al. did not observe improvements in the OS of patients with eBDC using a higher dose of BED > 59.5 Gy10 to segments of tumor distal from the small bowel vs. conventional external beam radiotherapy to a BED ≤ 59.5 Gy10 [29]. These results partly demonstrated that the irradiated dose threshold, BED ≤ 59.5 Gy10, is lower than the BED 80.5 Gy10 used in Tan’s study, and may not be sufficient to control the tumor. In SBRT, Brunner et al. also found that OS was significantly improved after higher dose irradiation (BED max 91 Gy inside the tumor) for eBDC [30]; our data concurred with Brunner’s findings, and could widen the potential of PT, which is an option to prescribe higher doses to improve outcomes for them.

We identified GTV as a significant predicator for survival, but only in eBDC and not in iBDC. Brunnner et al. reported that significance of tumor diameter 40 mm at diagnosis distinguished two survival profiles (21.4 vs. 8.7 months; *p* = 0.01) in non-bulky eBDC treated with chemoradiotherapy using conventional 3D-CRT [31]. On the contrary, however, tumor size and PTV were neither predictive nor prognostic for LC and OS for SBRT, treating mix population with eBDC and iBDC by the same author [30]. There is a controversy with pros [9,29] and cons [12] for importance of tumor volume for survival, and therefore this issue should be left for further explorations.

Baseline CEA levels were also identified as significant predicators for survival. This is natural because the baseline CEA level is one of the most universally used blood tumor marker, which impacts survival in several cancers, including BDC [1,5].

This study has several limitations. First, retrospective multicenter data accumulation is prone to selection bias, which may compromise the completeness of data, especially on late toxicity. Second, the lack of histological confirmation. Despite combined brush cytology and forceps biopsy, there were certain difficulties in obtaining histological confirmation [32]. In fact, we could not find a statistical difference in OS between patients who were histopathogically diagnosed (2-year overall survival rate of 46.9%) and patients with imaging and tumor markers (43.9%). Third, although we could not find a role for systemic therapy, several new systemic treatments may influence the outcome. Despite these limitations, this multicenter study is one of the largest analyses of BDC.

## 5. Conclusions

In conclusion, this multicenter study showed good efficacy with a low incidence of severe toxicity of PT in patients with BDC, both eBDC and iBDC, who did not undergo surgery.

## Figures and Tables

**Table 1:** Patient characteristics in total population and each location of tumor.

Variables	Strata	Total (*n* = 150)	eBDC (*n* = 82)	iBDC (*n* = 68)	*p*-Value
		No. (%) or Median (range)			
Age		74.00 (50.00, 94.00)	76.00 (53.00, 92.00)	73.50 (50.00, 94.00)	0.488
Gender	Female	53 (35.3)	25 (36.8)	28 (34.1)	0.864
	Male	97 (64.6)	43 (63.2)	54 (65.9)	
Performance status	0	120 (80.0)	62 (75.6)	58 (85.3)	0.579
	1	21 (14.0)	14 (17.1)	7 (10.3)	
	2	6 (4.0)	4 (4.9)	2 (2.9)	
	3	3 (2.0)	2 (2.4)	1 (1.5)	
Child-Pugh class	Normal-A	137 (91.3)	76 (92.7)	61 (89.7)	0.430
	B	12 (8.0)	5 (6.1)	7 (10.3)	
	C	1 (0.7)	1 (1.2)	0 (0.0)	
Diagnosis	Pathological	66 (44.0)	42 (51.2)	24 (35.3)	0.069
	Imaging+ tumor markers	84 (56.0)	40 (48.8)	44 (64.7)	
Jaundice	No	97 (64.7)	40 (48.8)	57 (83.8)	**<0.001**
	Yes	53 (35.3)	42 (51.2)	11 (16.2)	
Operability	No	134 (89.3)	73 (89.0)	61 (89.7)	1
	Yes	16 (10.7)	9 (11.0)	7 (10.3)	
Proton or Carbon	Proton	140 (98.7)	82 (100)	66 (97.1)	0.39
	Carbon	2 (1.3)	0 (0.0)	2 (2.9)	
Vascular invasion	No	69 (46.0)	42 (51.2)	27 (39.7)	0.189
	Yes	81 (54.0)	40 (48.8)	41 (60.3)	
Primary location	iBDC	68 (45.3)	-	68 (100)	NA
	eBDC: Perihilar	56 (37.3)	56 (68.3)	-	
	Gallbladder	9 (6.0)	9 (11.0)	-	
	Distal	17 (11.3)	17 (20.7)	-	
T category	1	NA	Hilar:GB:Distal = 3:0:7	22 (32.8)	NA
	2	NA	Hilar:GB:Distal = 15:1:6	28 (41.8)	
	3	NA	Hilar:GB:Distal = 10:6:3	7 (10.4)	
	4	NA	Hilar:GB:Distal = 28:2:1	10 (14.9)	
N category	0	118 (78.7)	59 (72.0)	59 (86.8)	**0.03**
	1	32 (21.3)	23 (28.0)	9 (13.2)	
Tumor size (diameter)	cm^3^	4.00 (1.00, 15.30)	3.00 (1.00, 9.00)	5.05 (1.00, 15.30)	**<0.001**
GTV	cm^3^	31.87 (0.00, 1526.00)	18.66 (0.00, 467.06)	63.70 (1.28, 1526.00)	**<0.001**
CTV	cm^3^	79.99 (9.80, 1526.00)	57.39 (11.72, 588.62)	117.28 (9.80, 1526.00)	**<0.001**
Distance between tumor and intestine	<1 cm	85 (56.7)	63 (76.8)	22 (32.4)	**<0.001**
	≥1 cm	65 (43.3)	19 (23.2)	46 (67.6)	
Pre-RT chemotherapy	No	93 (62.0)	47 (57.3)	46 (67.6)	0.238
	Yes	57 (38.0)	35 (42.7)	22 (32.4)	
Concurrent chemotherapy	No	74 (49.3)	42 (51.2)	52 (76.5)	**0.002**
	Yes	56 (37.3)	40 (48.8)	16 (23.5)	
Post-RT chemotherapy	No	85 (56.7)	43 (52.4)	42 (61.8)	0.193
	Yes	51 (34.0)	33 (40.2)	18 (26.5)	
	Unknown	14 (9.3)	6 (7.3)	8 (11.8)	
Baseline CEA level	ng/mL	3.65 (<0.50, 3807.60)	3.35 (<0.50, 79.40)	4.00 (<0.60, 3807.60)	0.073
Radiation dose	GyRBE	70.20 (44.00, 77.00)	70.00 (44.00, 77.00)	72.60 (60.00, 77.00)	**<0.001**
Number of fractions	fr	25.00 (10.00, 38.00)	26.00 (20.00, 38.00)	22.00 (10.00, 38.00)	**<0.001**
Prescribed dose in EQD2	Gy	76.00 (44.00, 91.30)	72.92 (44.00, 87.40)	80.47 (70.00, 91.30)	**<0.001**

Bold values indicate statistically significance, Equivalent 2-Gy fractions: EQD2 = *n* × *d* × ((α/β) + d)/((α/β) +2): *n* = number of treatment fractions: *d* = dose per fraction in Gy, α/β = 10, eBDC = extrahepatic biliary duct carcinoma, iBDC = intrahepatic biliary duct carcinoma, CEA = carcinoembryonic antigen, NA = not available.

**Table 2:** Uni- and multivariate analysis for overall survival rate using Cox proportional hazards model.

Variable	Strata	Univariate Analysis	Multivariate Analysis	
		*p*-Value	Hazard Ratio (95% CI)	*p*-Value
Age	Sequential	0.88		
Gender	Male vs. Female	0.77		
Performance status	0–1 vs. 2–3	0.301		
Location	iBDC vs. eBDC	0.68		
Operability	No vs. Yes	0.282		
Diagnosis	Pathological vs. others	0.928		
Jaundice	No vs. Yes	0.974		
N category	0 vs. 1	0.77		
Tumor diameter	<6.3 cm vs. 6.3 cm≤	0.136	1.66 (0.86–3.20)	0.129
GTV	<28 cm^3^ vs. 28 cm^3^≤	0.3		
CTV	<75 cm^3^ vs. 75 cm^3^≤	0.299		
Vascular invasion	No vs. Yes	0.0668	2.26 (1.24–4.11)	**0.007**
Baseline CEA level	<5 ng/mL vs. 5 ng/mL≤	**0.0264**	3.18 (1.83–5.52)	**<0.0001**
Distance from GI	<1 cm vs. 1 cm≤	0.2696		
Radiation dose in EQD2	EQD2 < 67 Gy vs. EQD2 ≥ 67 Gy	**0.0356**	0.371 (0.19–0.72)	**0.003**
Chemotherapy	No vs. Yes	0.5849		
	Neoadjuvant	0.3315		
	Concurrent	0.3652		
	Adjuvant	0.3315		

Bold values indicate statistically significance. Abbreviations; CI = confidence interval, CEA = carcinoembryonic antigen, eBDC = extrahepatic biliary duct carcinoma, iBDC = intrahepatic biliary duct carcinoma.

**Table 3:** Pattern of failure after particle beam therapy.

Status	All Patients	(%)	iBDC	eBDC
Alive, no progression	29	(19.3%)	17	23
Progression	80	(53.3%)	39	41
Local failure (inside radiation field)	29	(19.3%)	13	16
Intrahepatic failure outside irradiated field	29	(19.3%)	16	13
Lymph node	12	(8.0%)	7	5
Distant metastasis	26	(17.3%)	12	14
Detail place of distant metastases			Lung 10, Peritoneum 2, Bone 1, Submental Lymph Node 1	Lung 4, Bone 2, Peritoneum 6, Abdominal wall 2, Pleural 1, Rectum 1
Alive with disease progression	35	(23.3%)	18	17
Dead of disease with progression	45	(30.0%)	21	24
Dead of other causes, no progression	29	(19.3%)	11	18

Number of patients does not equal number of progressions, as several patients showed multiple progression sites. eBDC = extrahepatic biliary duct carcinoma, iBDC = intrahepatic biliary duct carcinoma.

**Table 4:** Uni- and multivariate analysis for overall survival rate using Cox proportional hazards model in eBDC.

Variable	Strata	Univariate Analysis	Multivariate Analysis	
		*p*-Value	Hazard Ratio (95% CI)	*p*-Value
Age	Sequential	0.33		
Gender	Male vs. Female	0.83		
Performance status	0–1 vs. 2–3	0.85		
Operability	No vs. Yes	0.346		
Diagnosis	Pathological vs. others	0.406		
Jaundice	No vs. Yes	0.957		
N category	0 vs. 1	0.914		
Tumor diameter	<6.3 cm vs. 6.3 cm≤	0.194		
GTV	<12 cm^3^ vs. 12 cm^3^≤	0.075	2.30 (1.14–4.66)	**0.01**
CTV	<75 cm^3^ vs. 75 cm^3^≤	0.371		
Vascular invasion	No vs. Yes	0.354		
Baseline CEA level	<5 ng/mL vs. 5 ng/mL≤	0.097	1.55 (0.76–3.15)	0.219
Distance from GI	<1 cm vs. 1 cm≤	0.776		
Radiation dose in EQD2	EQD2 < 67 Gy vs. EQD2 ≥ 67 Gy	**0.030**	0.45 (0.24–0.87)	**0.018**
Chemotherapy	No vs. Yes	0.850		
	Neoadjuvant	0.351		
	Concurrent	0.312		
	Adjuvant	0.125	0.57(0.29–1.12)	**0.10**

Bold values indicate statistically significance.

**Table 5:** Uni- and multivariate analysis for overall survival rate using Cox proportional hazards model in iBDC.

Variable	Strata	Univariate Analysis	Multivariate Analysis	
		*p*-Value	Hazard Ratio (95% CI)	*p*-Value
Age	Sequential	0.277		
Gender	Male vs. Female	0.459		
Performance status	0–1 vs. 2–3	0.906		
Operability	No vs. Yes	0.564		
Diagnosis	Pathological vs. others	0.441		
Jaundice	No vs. Yes	0.644		
N category	0 vs. 1	0.597		
Tumor diameter	<6.3 cm vs. 6.3 cm≤	0.597		
GTV	<28 cm^3^ vs. 28 cm^3^≤	0.67		
CTV	<75 cm^3^ vs. 75 cm^3^≤	0.313		
Vascular invasion	No vs. Yes	0.074	2.15 (0.95–4.87)	**0.065**
Baseline CEA level	<5 ng/mL vs. 5 ng/mL≤	**0.0003**	4.08 (1.93–8.63)	**0.0002**
Distance from GI	<1 cm vs. 1 cm≤	0.295		
Radiation dose in EQD2	EQD2 < 67 Gy vs. EQD2 ≥ 67 Gy	**NA**		
Chemotherapy	No vs. Yes	0.3926		
	Neoadjuvant	0.273		
	Concurrent	0.623		
	Adjuvant	0.273		

Bold values indicate statistically significance.

**Table 6:** Toxicity grade 3 or more after particle beam therapy.

Location	Toxicity	Acute Toxicity Grade ≥ 3 PT NO (%)	Late Toxicity Grade ≥ 3 PT NO (%)
Gastrointestinal	Duodenal perforation		1 (1.1%)
	Duodenal bleeding		2 (1.3%)
Bile duct	Bile duct stenosis	2 (2.2%)	1 (1.1%)
Liver	Failure		1 (1.1%)
Total		2 (2.2%)	4* (2.7%)

## Data Availability

The data of this study be obtained from the author upon reasonable request.

## Supplementary Materials

The following supporting information can be downloaded at: https://www.mdpi.com/article/10.3390/cancers14235864/s1, Figure S1: (A) Local control rate according to CTV, (B) Local control rate according to EQD2, (C) Progression free survival rate according to CEA level; Table S1, Detailed treatment schedule; Table S2: Comparison of background characteristics in patients treated with carbon and proton.; Table S3. Uni- and Multivariate analysis for local control rate using Cox proportional hazards model.; Table S4. Uni- and Multivariate analysis for progression free survival rate using Cox proportional hazards model.
