# Supplementary material for: Particle Beam Therapy for Intrahepatic and Extrahepatic Biliary Duct Carcinoma: A Multi-Institutional Retrospective Data Analysis
Source: Cancers (Basel). 2022 Nov 28;14(23):5864. doi: 10.3390/cancers14235864 (PMC9736951; doi:10.3390/cancers14235864)
Supplement: Supplementary file 1 [file cancers-14-05864-s001.zip › supplemental Figures_cancers.pptx]

## Slide 1
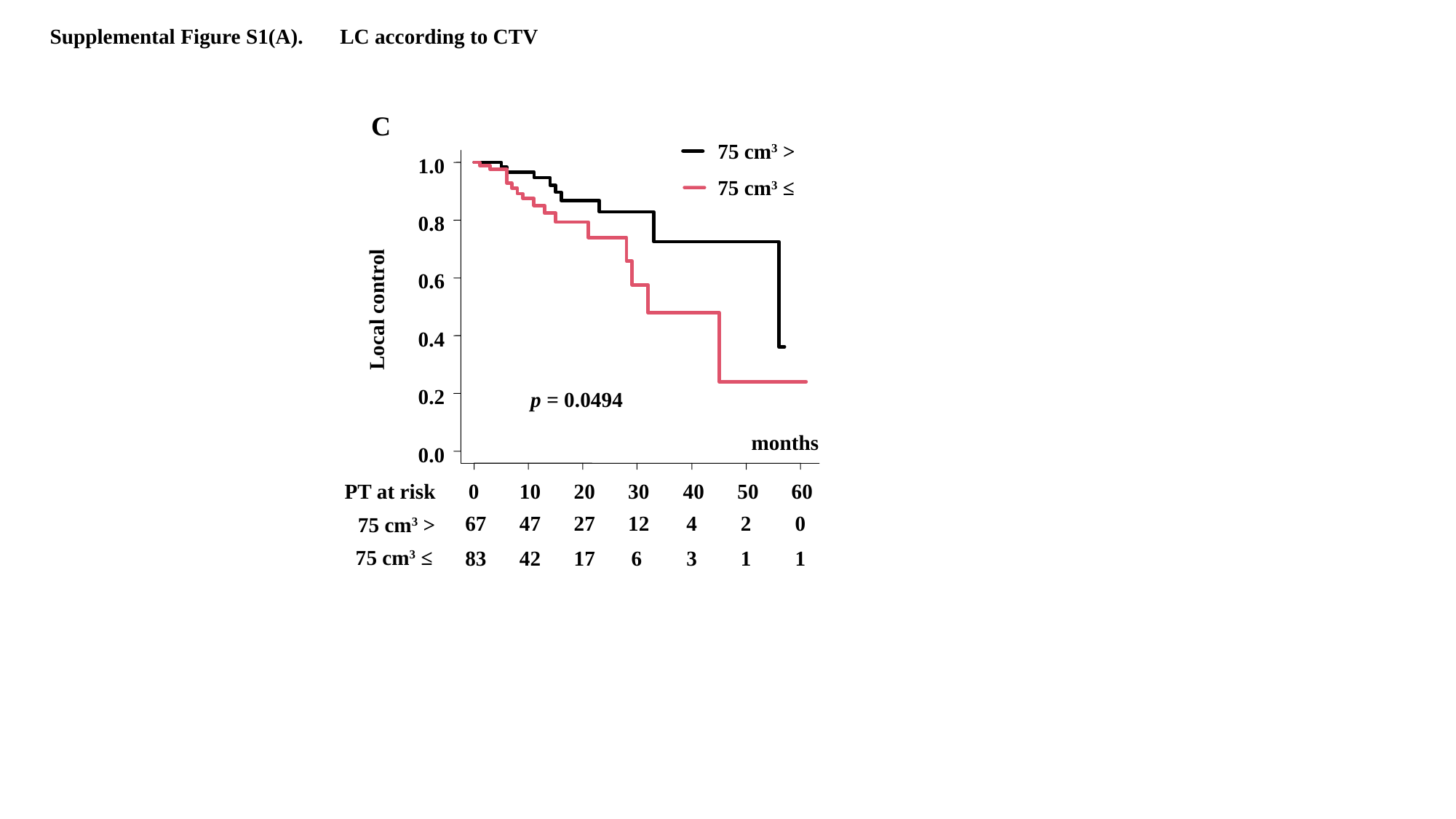

Supplemental Figure S1(A). 　LC according to CTV
C
75 cm3 >
1.0
75 cm3 ≤
0.8
0.6
Local control
0.4
p = 0.0494
0.2
months
0.0
0
10
20
30
40
50
60
PT at risk
67
47
27
12
4
2
0
75 cm3 >
75 cm3 ≤
83
42
17
6
3
1
1

## Slide 2
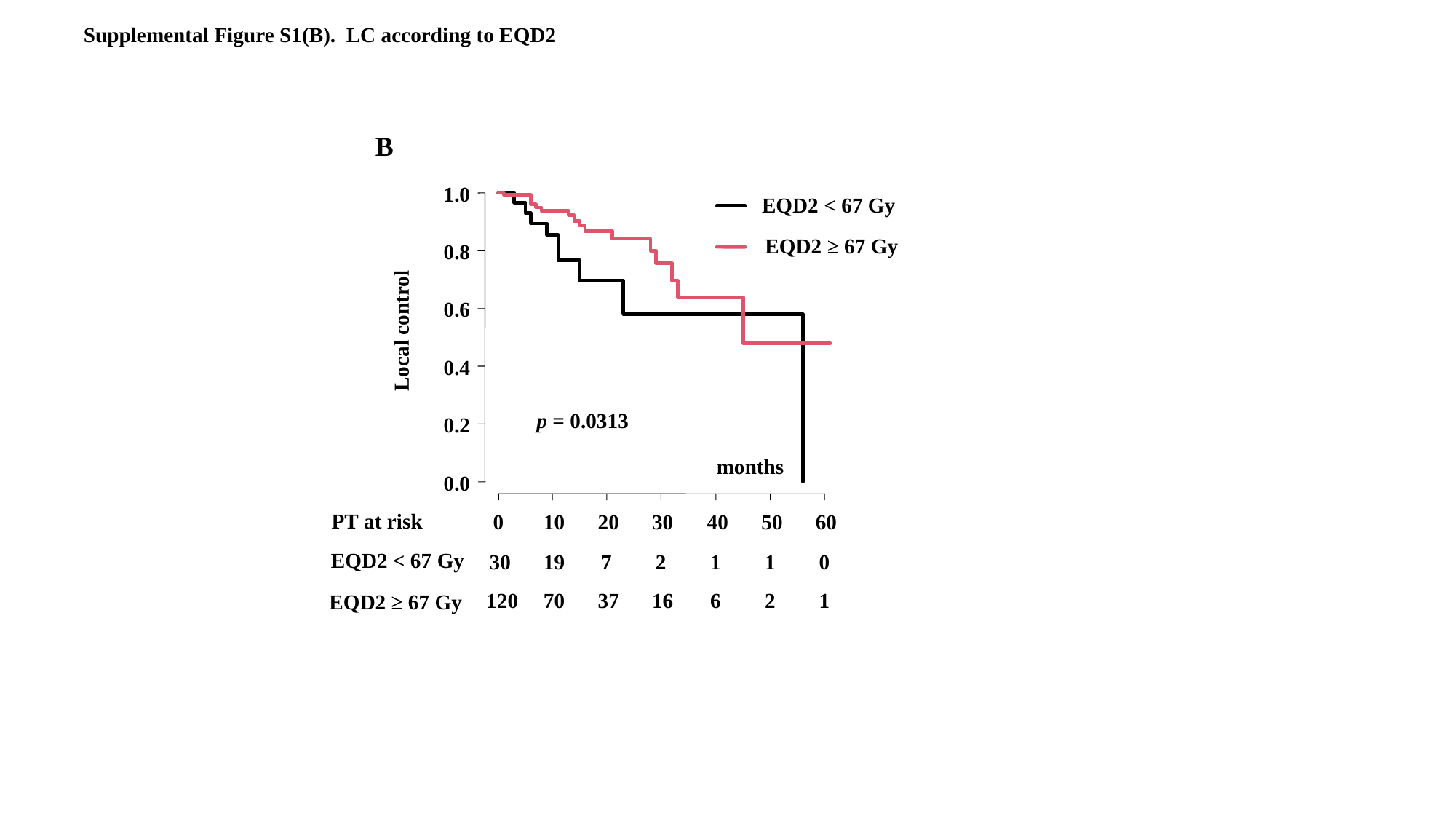

Supplemental Figure S1(B). LC according to EQD2
B
1.0
EQD2 < 67 Gy
EQD2 ≥ 67 Gy
0.8
0.6
Local control
0.4
p = 0.0313
0.2
months
0.0
PT at risk
0
10
20
30
40
50
60
EQD2 < 67 Gy
30
19
7
2
1
1
0
EQD2 ≥ 67 Gy
120
70
37
16
6
2
1

## Slide 3
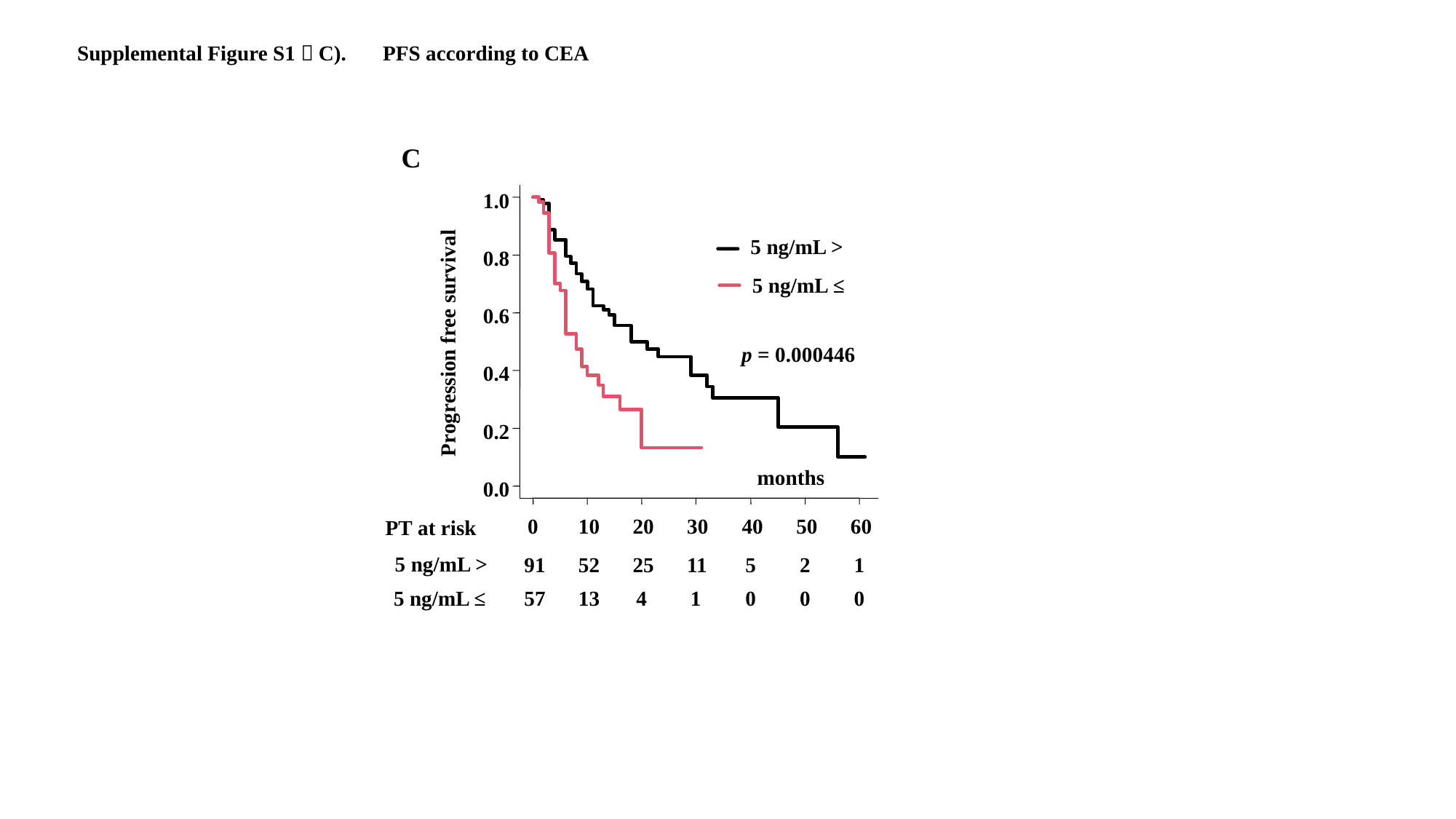

Supplemental Figure S1（C). 　PFS according to CEA
C
1.0
5 ng/mL >
0.8
5 ng/mL ≤
0.6
Progression free survival
p = 0.000446
0.4
0.2
months
0.0
0
10
20
30
40
50
60
5 ng/mL >
91
52
25
11
5
2
1
5 ng/mL ≤
57
13
4
1
0
0
0
PT at risk
